# Telehealth and Technology for Diabetes in Pregnancy Clinics: Staff Perspectives from South Auckland, New Zealand

**DOI:** 10.1155/2024/6429519

**Published:** 2024-03-14

**Authors:** Megan Singhal, Charlotte Oyston

**Affiliations:** ^1^Department of Obstetrics and Gynaecology, Faculty of Medical and Health Sciences, University of Auckland, Auckland, New Zealand; ^2^Department of Obstetrics and Gynaecology, Middlemore Hospital, Te Whatu Ora Counties Manukau Health, South Auckland, New Zealand

## Abstract

Providing care for patients with diabetes in pregnancy (DiP) provides unique challenges beyond those faced in standard antenatal care or diabetes outside of pregnancy. Teleclinics (use of telephone, email, or other technologies) as an alternative to in-person clinic appointments have become more widely used for care since the start of the COVID-19 pandemic. To understand how teleclinics might be improved for ongoing use, it is important to understand the experiences and perceptions of the clinicians involved in DiP care. *Aim*. To understand staff experiences of DiP teleclinics and gain their perspectives on if and how teleclinics and other technologies might be best used in the future. *Methods*. A qualitative study using semistructured interviews of healthcare providers in a large DiP service. Twenty staff members (midwifery, obstetrics, physician, dietician, and administration) were approached to participate. Fifteen staff across 5 specialties consented to be interviewed. Template analysis of interview transcripts was performed, with a focus on 3 themes: collaboration and working together are important for providing care for DiP, a need for flexibility in scheduling and the ability to individualise the way care is provided, and challenges to adapting to new technology. *Results*. Potential benefits of teleclinics were acknowledged, but respondents also viewed teleclinics as not suitable for all DiP patients due to different needs and risks. Challenges to using teleclinics include establishing good rapport and the current limited infrastructure and patient resources. *Conclusion*. Healthcare providers viewed teleclinics as a way of supporting rather than replacing current care. Maintaining flexibility in clinic scheduling to allow incorporation of teleclinics into patient's current schedule of visits *ad hoc* and providing extra technical and administrative support are important considerations for developing a teleclinic service.

## 1. Introduction

Diabetes in pregnancy (DiP) encompasses both gestational (first recognition or onset in pregnancy, with resolution following birth) and preexisting (type 1 or 2) diabetes. Hyperglycaemia is associated with poorer pregnancy and childhood outcomes. Achieving glycaemic control is essential to reducing the risk of developing complications such as preeclampsia, macrosomia, and perinatal mortality [1]. Providing accessible and timely care for patients with DiP is a source of increasing pressure on healthcare systems due to increasing rates of DiP worldwide [2]. In New Zealand, 61,000 people give birth annually, 4.9% of whom have pregnancies complicated by diabetes. In particular, the rate of diabetes in pregnancy is increasing amongst people of South Asian, Pacific Island, and Māori ethnicities [3]. In our tertiary, public hospital in South Auckland, New Zealand, while rates of births have remained (7,000 births/year), rates of DiP have increased from 3% to 12% of births between 2006 and 2019 [4].

Managing diabetes during pregnancy has unique complexities beyond providing healthcare during uncomplicated pregnancy or managing diabetes outside of pregnancy. Compared to nonpregnant individuals, targets for glycaemic control are lower, and insulin resistance increases as gestation advances, meaning uptitration of medications over a short timeframe may be required to maintain control. Furthermore, most cases of DiP are gestational diabetes, where the diagnosis is made at 24-28 weeks' gestation (term = 40 weeks' gestation) [5–7]. There is a short timeframe for patients to come to terms with their diagnosis, learn and adapt to regular blood sugar testing and treatment, and make modifications to their lifestyle. As opposed to diabetes outside pregnancy, DiP care requires multidisciplinary support that adapts to the needs of individuals within short timeframes [8].

Teleclinics (using any or a combination of web, email, mobile, or app-based technology to provide health services remotely) is a means of providing care for DiP. Although previously underutilised, teleclinics have become the default method of providing healthcare in many areas during the COVID-19 pandemic [9–12]. In our hospital during the COVID-19 pandemic, we utilised predominately telephone-based clinics, with some clinicians using email, text messaging, and video calling to supplement care. We surveyed patients who had used a DiP teleclinic and found that those surveyed were highly satisfied with this method of consultation and that teleclinics may be useful for overcoming some barriers to accessing care [13]. Due to the growing pressures on healthcare systems, the need for workplace flexibility, and the time-sensitive and time-intensive monitoring provided for DiP, we were interested in leveraging the experiences of teleclinics during the COVID-19 pandemic to better understand how clinics could be optimised for DiP.

The aim of the present study was to understand staff experiences of teleclinics and/or other technologies for DiP care and to gain staff perspectives on if and how teleclinics might be best used in the future, compared to or alongside in-person clinics.

## 2. Materials and Methods

This is a qualitative analysis of semistructured interviews with clinicians involved in DiP Care at Te Whatu Ora Counties Manukau, South Auckland, New Zealand.

Ethical approval was obtained from the Auckland Health Research Ethics Committee on 2 June 2021 (AH 22265).

### 2.1. Setting

The setting of this study was a single centre tertiary level public hospital in South Auckland, New Zealand. At the time of the study, the DiP service at Counties Manukau Health reviewed approximately 800-900 patients a year. This service is run by 7 specialist DiP midwives (patients also have their own independent midwife), 6 specialist obstetricians, 5 community health workers, 3 physician/endocrinologists, 2 dieticians, and 2 nonclinical support staff.

### 2.2. Recruitment

Between 10 January 2022 and 14 February 2022, staff who provided care for DiP patients, including administrators, dietitians, DiP midwives, obstetricians, and physicians, were approached by email to participate. Inclusion criteria were people who were employed in the DiP service, having at least one-year experience providing care through the clinic, being able to understand/communicate in English, and availability during the study period. Those who were interested were emailed the participant information sheet and an electronic consent form. Our purpose was to provide a rich description from diverse viewpoints rather than make comparisons between different specialties or characteristics of the participants such as age or ethnicity. To ensure a broad range of perspectives from health providers, we aimed for a total sample of 15 clinical staff, including more than 1 from each specialty across midwifery, physicians, obstetrics, dieticians, and administration. The sample size was determined by the availability of participants and resources for the completion of the study.

### 2.3. Data Collection

A qualitative exploratory study design was chosen to gain a broad understanding and a range of perspectives from those involved in the DiP service. To aid in collecting a diverse range of answers, semistructured interviews were conducted. Semistructured interviews were carried out one-on-one or in a group setting and in-person or over the phone/zoom, depending on the interviewee's preference. The interviews were conducted with the aid of a guide (see guide in the supplementary material (available here)). One specialty (midwifery) opted to carry out the interview together as a group, while all other participants preferred one-on-one interviews.

### 2.4. Analysis

Interviews were audio-recorded and transcribed verbatim. The audio recordings and transcripts were stored in a password protected file and were anonymised to ensure patient confidentiality. The transcripts were analysed using a template approach described by King and Brooks [14]. Each author had a copy of the transcripts and undertook line-by-line readings of the transcript independently. Handwritten notes were made regarding thoughts, impressions, and important aspects of parts of the transcripts. Following careful rereading of the transcripts, each section of the transcript was assigned labels or codes to summarise the essence of the text. Similar codes were then grouped together into meaningful clusters and organised into themes. Within each theme, codes were ordered hierarchically, with the highest order codes being the broadest and lower ordered codes representing more focussed themes within a broader theme. The initial coding template was discussed and developed by both authors using a table on Microsoft Excel. The two authors reviewed all transcripts to determine whether themes defined in the initial template adequately represented the data. Where the specified themes did not readily fit the data, the template was reviewed, discussed, and revised as necessary. The described themes were modified, refined, and agreed upon by both authors with repeated review and reading of transcripts until the authors felt the themes represented a full description of the data relevant to the study's research question. The themes are described, with narrative examples provided for each theme, accompanied by the professional role of the participant.

## 3. Results

Twenty health providers in the DiP service were approached to participate in the study. Five did not respond, and the remaining 15 people participated in this study (response rate of 75%). This included 5 DiP midwives, 5 obstetricians, 2 physicians, 2 dieticians, and 1 administrative staff member. 13 out of the 15 participants were female, and experiences in the local service ranged from 1 to over 10 years.

Following the review of the transcripts, a template was constructed consisting of three themes:

1. Collaboration and working together are important for providing care for DiP
2. There is a need for flexibility in scheduling and the ability of individualising care
3. Challenges to adapting to new technology

### 3.1. Theme 1: Collaboration and Working Together Are Important for Providing Care for DiP

Amongst all professions, a collaborative relationship with patients was identified as being essential to good care. This required trust and the ability to build rapport. Subthemes within this theme were that teleclinics had the potential to both positively and negatively impact relationships and the ability to work collaboratively (Table 1) and that the addition of video calling might also positively or negatively affect this relationship (Table 2).

Some respondents felt that teleclinics facilitated greater engagement with patients. If patients were in the comfort of their own space, they would be more comfortable communicating freely with their healthcare providers, allowing them to work together more openly and effectively. Some interviewees felt that newer technologies or social media could provide enhanced two-way communication and partnership between the patients and their clinicians. Some interviewees expressed the opposite sentiment and felt that telephone consultation had negatively impacted their ability to build relationships with patients. Common reasons for this were the absence of nonverbal communication, difficulty maintaining rapport, increased distractibility, and lack of privacy over the phone compared to in-person consultations.

Replacing or supplementing telephone consultations with video conferencing, such as Zoom, might overcome the shortcomings of using a telephone, particularly when building rapport, educating patients, and troubleshooting problems. Alternatively, others voiced that implementing video conferencing would hinder access to care for patients who had limited access to internet data and mobile phones. The South Auckland population has a high proportion of Māori and Pacific women who experience a high level of deprivation compared to other ethnicities. There was concern that unless there were ways of ensuring all patients had good access to the technology, routinely offering appointments via videoconferencing might unintendedly privilege the service towards people of non-Māori and non-Pacific ethnicity, hence potentially exacerbating existing health inequities.

### 3.2. Theme 2: Need for Flexibility and Individualising Care in Clinics

In this theme, a one-size-fits-all approach to clinic scheduling was considered unworkable by all professions. Interviewees described a need for clinic appointments to accommodate patients with a diversity of clinical and socioeconomic needs. This required flexibility in the timing and duration of scheduled appointments and which staff patients were scheduled to see. Subthemes included determining which patients would be more suitable for face-to-face over teleclinic care and the need for a flexible/hybrid system, where teleclinics were incorporated alongside, rather than replace face-to-face visits.

#### 3.2.1. Who Would Be More Suitable for Face-to-Face Care?

Amongst all professions, there was concern about teleclinics inequitably disadvantaging groups, and that teleclinics would not adequately cater to the needs of medically complex patients, people with hearing loss or people who communicated via translator (Table 3). Both obstetricians and physicians were particularly concerned about the loss of opportunity to physically examine patients (blood pressure, capillary blood sugar testing, and obstetric palpating) over the phone. This was important, as there was a perception that patients in the South Auckland population could be medically complex with preexisting health conditions and low utilisation of healthcare. There was also concern about incomplete assessments in high-risk patients with multiple comorbidities who needed more intensive input in their care. Therefore, the face-to-face visits were an opportunity to optimise health issues not directly related to pregnancy. As English is the only common language spoken amongst clinic staff, non-English speaking patients are currently provided with a translator. This service was better accommodated for face-to-face in a clinic as clinicians faced difficulties with communication and coordinating with multiple people over the phone.

Midwives, physicians, and obstetricians believed that those most suitable patients for teleclinics included those with gestational (as opposed to type 1 or 2) diabetes, on a stable dose of metformin and those who were well-engaged/well-resourced patients whose main barriers to attending were time constraints due to childcare responsibilities and work commitments. For these individuals, it was felt that teleclinics would be a more suitable option for follow-up appointments. Teleclinics could help to increase accessibility for these individuals to attending DiP clinics by offering a more convenient mode of delivery of care.

#### 3.2.2. Need for Flexibility in Clinics

Interviewees described teleclinics as potentially improving flexibility if they could be incorporated alongside (as opposed to replacing) face-to-face visits. All respondents stated that regardless of the patient's situation, the first appointment should be conducted face-to face to introduce the service, educate, and build rapport. Factors such as type of diabetes (type 1 vs. type 2 vs. gestational), presence of other social support networks, and other needs or barriers that the patient experiences would determine suitability for future teleclinic appointments. Furthermore, the flexibility of teleclinics was considered as a way that could improve the attractiveness of the service to staff: “Telehealth would give the flexibility, and the service will be able to attract people to come back to work for us” and “…gives them the kind of opportunity to juggle that family-work balance.”

### 3.3. Theme 3: Implementing Increased Use of Technology/Teleclinics Will Require Extra Resources

The final theme describes a perceived need for extra support and resources due to the added complexity or learning curve associated with changing technology. Interviewees stated that teleclinics or more technology could not be added into the current service without extra resources. This included extra staff hours, physical hardware, and support staff (Table 4).

Teleclinic consultations were reported as being more time-consuming than in-person visits as they could require multiple attempts to contact the patient, and there was more digital or online administration such as completing prescription/ultrasound/laboratory request forms and completing consultation notes after the teleclinic. There was a perception of more digital administration when consultations were done remotely. There was a mixed opinion about whether this was more, or less efficient than paper-based systems; however, the move towards increased technology and teleclinics, in general, was felt to be “inevitable.” Further improvement of the current infrastructure was suggested to help support its use. Additional administrative staff, equipment upgrades, and staff training were viewed as necessities for making technology more user-friendly.

## 4. Discussion

Diabetes is a serious and common pregnancy complication where patients have time-dependent and often complex needs. Teleclinics might provide a means of increasing access and flexibility to care. In our service [13] and others [1, 15, 16], patients have expressed satisfaction with teleclinics for DiP. We believe there is potential for its use to improve flexibility and efficiency within our service. However, there is limited evidence on the views and needs of multidisciplinary health providers to guide how services might be optimised. Based on our results, we have made the following recommendations, both for our own clinic and others trying to develop similar services.

(1) Teleclinics should be used to supplement rather than replace face-to-face care
  - The first appointment is offered face to face
  - Maintain flexibility in the allocation of face-to-face/teleclinic appointments, so that each appointment can be decided in advance in a shared patient and clinician decision-making framework
(2) Service-wide standardised criteria should be developed to determine which patients are most suitable to offer teleclinics to
(3) Consider the implementation of video calling
  - Provide support for all patients and staff who would be eligible for video calling appointments with appropriate technology
(4) Provide dedicated onsite IT support for staff, with high-quality hardware and software provided for both patients and staff members

### 4.1. Recommendation 1: Teleclinics Be Used to Supplement, Rather than Replace Face-to-Face Care

Like those interviewed in our study, in other studies, DiP clinicians, general obstetricians, and primary care physicians expressed that teleclinics could not replace but rather act as a complement to face-to-face care [1, 15–20]. These studies report that the benefits of virtual DiP consultations for suitable individuals include increasing the capacity to meet increased demands on the service while allowing complex consultations to be done face-to-face. Concerns of reduced rapport, inability for physical examination, language barriers, and patient access to adequate technology were consistent with our study. To balance these considerations, our clinic needs to be flexible with offering both teleclinics and face-to-face appointments. Having the first appointment face-to-face facilitates rapport building and acquiring additional information about the patient's risk and needs. The following appointments may be then allocated to teleclinics or face-to-face appointments, depending on appropriateness.

### 4.2. Recommendation 2: Creation of Standardised Criteria

To ensure that teleclinics do not perpetuate existing health inequity, we suggest developing a standardised criteria considering the patient's risks and needs to help clinicians appropriately allocate teleclinic services to suitable patients. From individuals involved in our study, patients with preexisting diabetes, other complex comorbidities (e.g., hypertensive disease, foetal growth restriction, and symptomatic cardiorespiratory disease), language barriers, and difficulty accessing the required technology would not be suitable for teleclinics. Other studies suggested similar considerations of patient's resource limitations, level of technology literacy, lack of privacy, and childcare that favour face-to-face consultations [1, 17, 21].

### 4.3. Recommendation 3: Implementation of Video Calling

Currently, our DiP clinic is limited to using telephone as its main method of teleclinic communication with patients. Our respondents voiced similar preferences to those previously reported about the benefits video consultations have compared to using only telephone [16, 21]. In studies, clinicians highlighted the benefits of video as having improved rapport compared to telephone, gaining insight into a patient's home environment and increased ability to involve family members during consultations [16, 18, 21, 22]. Collaborating and educating family members of DiP patients can be beneficial in achieving optimal glycaemic control [23].

Health providers in our own and other studies had concerns about the impact of video consultations on disadvantaged patients with affordability and technological barriers [20, 21, 24]. While audio-only virtual visits could be provided for these individuals, finding innovative ways to reduce barriers for patients accessing teleclinic appointments is necessary for equity. A possible option could be the implementation of local hubs that would provide resources and space for patients to attend their virtual appointments. A rural American study found high levels of satisfaction and acceptability for both patients and health providers when having an endocrinologist operating from an urban site while the diabetes patient visited a local hub with videoconferencing operated by a diabetes nurse [25]. Having a hub could help to reduce concerns clinicians in our study expressed about patients' access to a private, safe, and well-resourced place for teleclinic appointments. Having multiple hubs around South Auckland would meet the need for increased accessibility and convenience for patients. Hence, this alternative model could hugely benefit clinicians and patients alike, and we recommend that this should be investigated further.

### 4.4. Recommendation 4: Additional It Support with High-Quality Hardware and Software

Having adequate infrastructure to support teleclinic use by clinicians was another necessary consideration. Incompatible technology and inadequate IT administration support were challenges identified in our study and others [1, 15, 17]. These factors increase staff resistance and reluctance to change their existing practice [15]. Thus, making technology user-friendly with updated software and engaging with staff is important for the successful implementation of teleclinics. Creating new roles within the service was viewed as a means of accomplishing this. The main roles identified were teleclinic champions to educate/encourage staff to use teleclinics and IT coordinators to oversee and monitor the provision of support to clinicians [1, 17].

## 5. Strengths and Limitations

Despite the prevalence of DiP and a recent increase in the utilisation of teleclinics, there is limited data to describe service provider experience of teleclinics for DiP care. Although our study is small and conducted at a single centre, we believe the findings will be useful to others trying to develop their DiP service. We obtained a representative cross-section of specialties involved in DiP care and found a significant repetition of concepts during analysis, suggesting that sampling was adequate. Although the insights will be directly generalisable to our own service, our study described similar themes to those in other international studies. This suggests that our findings and recommendations will likely also be useful to other centres (including those outside New Zealand) considering ways of developing their teleclinic service for DiP.

## 6. Conclusions

DiP service providers believe that teleclinics/teleclinic appointments for DiP are potentially a useful way to provide care but that they are not suitable for all patients at all points in their pregnancy due to different needs. Teleclinics may facilitate collaboration and flexibility of appointments, which may improve care for some. Developing a way of integrating teleclinics alongside standard/face-to-face and ensuring adequate support for staff and patients are important factors in determining the success and usefulness of teleclinics.

## Figures and Tables

**Table 1:** The impact of teleclinics on the ability to work collaboratively with patients.

Potential positive impacts of teleclinics
“Sometimes, if the women aren't face-to-face...there's a bit of anonymity, you can sometimes get more from them.” (Midwife) “Some people might feel intimidated face-to-face in front of people but feel a bit more empowered when they are…at home or with their whānau (family).” (Obstetrician)
Potential negative impacts of teleclinics
“You cannot really build rapport with a disembodied voice.” (Physician) “You cannot see whether or not they are engaged in the conversation or are they just saying ‘yes, doctor.'” (Obstetrician) “It takes longer to describe more complex information, and you do not have nonverbal feedback quite as clearly as you would in a face-to-face setting.” (Obstetrician)

**Table 2:** The impact of video calling on the ability to work collaboratively with patients.

Video calling would be an asset
“.. for them (patients) to know who their doctor or midwife is, or her dietician is, is different when it's a voice versus a voice and a live video link.” (Physician) “Video consult would be better… you can easily gauge if someone understands…you can adjust how you are explaining something according to their reaction.” (Obstetrician) “... if they say, ‘I've got a problem with my meter', and if they can switch on their video and show me what they are having problems with, it's way easier than describing it to me.” (Dietitian)
Video calling would be a disadvantage
“The other barrier is economical. Most of our women live in the most deprived areas and therefore, do not have the credit on their phone or the data to be able to, you know, accept phone calls.” (Obstetrician) “…barriers may be concealed by face-to-face clinics. But, when it becomes a phone consult or video link, we may find that our patients will struggle with it. So, it's the question of availability, affordability, and ability to utilise and with that comes equity amongst our patients.” (Physician)

**Table 3:** Who would be more suitable for face-to-face over teleclinic care?

Teleclinics do not provide a complete clinical picture of medically complex patients
“(the patient) said that she was fine, but actually, she was having an asthma exacerbation…and her sugars were really crazy. Because of the COVID lockdown, the only food that she had access to was rice to feed her whole family, and actually, I think if you do not see a patient face-to-face sometimes, you cannot unpick all of that.” (Obstetrician) “We take the opportunity to do the CBG (capillary blood glucose) in clinic, and if it confirms out suspicious of (patients) writing these levels to please us or to avoid testing, then we can reveal that issue and try to address it in a respectful and constructive way.” (Physician) “But some (high-risk patients) do not show what's happening. They do not have the (capillary blood glucose) numbers with them, they do not send anything in…So you have to catch them face-to-face cause a single finger prick will tell you, this is a woman with a blood sugar of 15 and that I will be admitting them now.” (Physician)
Face-to-face visits are preferable when there are communication barriers
“A woman has been given a telephone appointment and I was thinking, I know English is her second language. You know, like she did understand some English, but I thought she was Tongan and a type 2 who does not engage very well. I cannot even get a hold of her by phone so I do not know how she got graded to be a telephone appointments…” (Midwife) “... it's really hard with an interpreter. Like I tried to do 3-way interpreter calls. It's doable, but it's really a struggle and for very superficial conversations…and it's another person where you are trying to their schedule align with, which is challenging as well.” (Obstetrician)
Teleclinics might improve access to care for patients who are not medically complex and who have other commitments
“Most of the women have got families and children…and they are sitting in clinic for two to four hours and they just cannot do that.” (Midwife) “…the lower risk women, the women that are diet-controlled or just on metformin and who have not got lots of other medical risk factors… so the suitability is just got to be for the lower risk.” (Obstetrician) “I found that telephone reviews have actually reduced the DNA (did not attend) rates, significantly. I think it's the whole, you know, getting to clinics, finding parking spots, waiting, whereas they know that they can just get on with life and expect a phone call from us.” (Dietitian)

**Table 4:** New technologies require extra resources and support to be implemented successfully.

New technologies alter the time and complexity of performing administrative tasks
“…you need to do prescriptions electronically- that takes double the time… you'd have to click it one by one, send it off, so all that doubles the time that you would otherwise take for a face-to-face (consultation).” (Physician) “We now have electronic requesting for ultrasounds and lab test … which we did not have (before). So that's all great and so much easier.” (Obstetrician)
Education, hardware, and additional support are needed
“(Using Zoom for diabetes education) was just too much. Like 3 computers later, trying to get internet access, asking people for help, logging on to different, oh my gosh, I mean it was so stressful that we kind of stopped.” (Midwife) “We're always struggling to find computers that work effectively… It's just a bit of a constant battle.” (Midwife) “…a little bit of education as it how it (technology) can work effectively…because we are a bit technologically challenged in some areas.” (Midwife) “We need people to do the job, not just technology. We need humans.” (Dietitian)

## Data Availability

The templates of coded data used to support the findings of this study are available from the corresponding author upon request.

## Supplementary Materials

Supplementary Materials A document containing a guide for interviews with participants was developed by the second author and field-tested and refined by the first author. It was used in all interviews and aided in exploring the participant's views of telehealth and technology in their practice of managing diabetes in pregnancy.

## References

[1] Rasekaba T., Nightingale G., Furler J., Lim W. K., Triay J., Blackberry I. (2021). Women, clinician and IT staff perspectives on telehealth for enhanced gestational diabetes mellitus management in an Australian rural/regional setting. *Rural and Remote Health*.

[2] Mastrogiannis D. S., Igwe E., Homko C. J. (2013). The role of telemedicine in the management of the pregnancy complicated by diabetes. *Current Diabetes Reports*.

[3] Jowitt L. M. (2016). Gestational diabetes in New Zealand ethnic groups. *Integrative Molecular Medicine*.

[4] Counties Manukau Health (2020). *Women’s Health and Newborn Annual Report 2019-2020*.

[5] Webber J., Charlton M., Johns N. (2015). Diabetes in pregnancy: management of diabetes and its complications from preconception to the postnatal period (NG3). *British Journal of Diabetes*.

[6] American Diabetes Association (2022). Standards of Medical Care in Diabetes-2022 abridged for primary care providers. *Clinical Diabetes*.

[7] Kampmann U., Knorr S., Fuglsang J., Ovesen P. (2019). Determinants of maternal insulin resistance during pregnancy: an updated overview. *Journal of Diabetes Research*.

[8] McElduff A., Cheung N. W., McIntyre H. D. (2005). The Australasian Diabetes in Pregnancy Society consensus guidelines for the management of type 1 and type 2 diabetes in relation to pregnancy. *Medical Journal of Australia*.

[9] Blandford A., Wesson J., Amalberti R., AlHazme R., Allwihan R. (2020). Opportunities and challenges for telehealth within, and beyond, a pandemic. *The Lancet Global Health*.

[10] Temesgen Z. M., DeSimone D. C., Mahmood M. (2020). Health care after the COVID-19 pandemic and the influence of telemedicine. *Mayo Clinic Proceedings*.

[11] Mann D., Chen J., Chunara R., Testa P., Nov O. (2020). COVID-19 transforms health care through telemedicine: evidence from the field. *Journal of the American Medical Informatics Association*.

[12] Perrin P., Pierce B. S., Elliott T. R. (2020). COVID-19 and telemedicine: a revolution in healthcare delivery is at hand. *Health Science Reports*.

[13] Shashikumar A., Okesene-Gafa K., Apaapa-Timu T. H., Wilson J., Oyston C. (2022). Teleclinics for the management of diabetes in pregnancy during COVID-19-maternal satisfaction and pregnancy outcomes. *New Zealand Medical Journal*.

[14] King N., Brooks J. (2018). Thematic analysis in organisational research. *The sage handbook of qualitative business and management research methods*.

[15] Given J., Bunting B., O’Kane M., Dunne F., Coates V. (2013). Tele-Mum: a feasibility study for a randomised controlled trail exploring the potential for telemedicine in the diabetes care of those with gestational diabetes. *Diabetes Technology and Therapeutics*.

[16] Zork N., Aubey J., Yates H. (2020). Conversion and optimization of telehealth in obstetric care during the COVID-19 pandemic. *Seminars in Perinatology*.

[17] Madden N., Emeruwa U., Friedman A. (2020). Telehealth uptake into prenatal care and provider attitudes during the COVID-19 pandemic in New York City: a quantitative and qualitative analysis. *American Journal of Perinatology*.

[18] Gomez T., Anaya Y. B., Shih K., Tarn B. (2021). A qualitative study of primary care physicians’ experiences with telemedicine during COVID-19. *The Journal of the American Board of Family Medicine*.

[19] Willcox J. C., van der Pligt P., Ball K. (2015). Views of women and health professionals on mHealth lifestyle interventions in pregnancy: a qualitative investigation. *JMIR mHealth and uHealth*.

[20] Wilson G., Currie O., Bidwell S. (2021). Empty waiting rooms: the New Zealand general practice experience with telehealth during the COVID-19 pandemic. *New Zealand Medical Journal*.

[21] Elawady A., Assaf A., Toure S., Cassidy C. (2020). Telemedicine during COVID-19: a survey of health care professionals’ perceptions. *Monaldi Archives for Chest Disease*.

[22] Brunton C., Arensberg M. B., Drawert S., Badaracco C., Everett W., McCauley S. M. (2021). Perspectives of registered dietitian nutritionists on adoption of telehealth for nutrition care during the COVID-19 pandemic. *Healthcare*.

[23] Martis R., Brown J., Crowther C. A. (2017). Views and experiences of New Zealand women with gestational diabetes in achieving glycaemic control targets: the views study. *Journal of Diabetes Research*.

[24] Almuslim H., AlDossary S. (2022). Models of incorporating telehealth into obstetric care during the COVID-19 pandemic, its benefits and barriers: a scoping review. *Telemedicine and e-Health*.

[25] Toledo F., Triola A., Ruppert K., Siminerio L. M. (2012). Telemedicine consultations: an alternative model to increase access to diabetes specialist care in underserved rural communities. *JMIR Research Protocols*.

[26] Singhal M., Oyston C. (2023). Staff perspectives of telehealth and technology for diabetes in pregnancy clinics in South Auckland, New Zealand. *Journal of Paediatrics and Child Health*.

