# Supplementary material for: Telehealth and Technology for Diabetes in Pregnancy Clinics: Staff Perspectives from South Auckland, New Zealand
Source: Int J Telemed Appl. 2024 Mar 14;2024:6429519. doi: 10.1155/2024/6429519 (PMC10957257; doi:10.1155/2024/6429519)
Supplement: Supplementary Materials — A document containing a guide for interviews with participants was developed by the second author and field-tested and refined by the first author. It was used in all interviews and aided in exploring the participant's views of telehealth and technology in their practice of managing diabetes in pregnancy. [file 6429519.f1.pdf]

Guide for interview with participants

1) To start with, could you please describe what you see as the most important parts of your job in caring for women with diabetes during their pregnancy?

    a) How do you think your role differs from the other health providers involved in the care of diabetic pregnant women?

    b) Is there anything about the current clinic that you think could be changed to help you carry out your role more effectively (or more enjoyably)?

2) We are interested in understanding whether some clinics could be done via telemedicine or virtually, but realise that there are some parts that might be more difficult or impossible. Are there any parts of the care you provide that you think are essential to be done face to face (ie that couldn't be done via teleclinics)?

    a) Why is this?

3) What parts (if there are there any) of the care you provide, do you think could be done well virtually or over the phone?

    a) Are there any virtual resources that you currently use to communicate to women with diabetes in pregnancy?

    b) Are there any virtual tools you would like to have/ that might be useful?

4) From a health provider's perspective, what are the potential barriers or difficulties to providing a telephone clinic for women with diabetes in pregnancy?

    a) Is there anything that you think could help reduce the barriers?

5) If teleclinics / virtual clinic were to become routine, would this be something you would like or consider being part of?

    a) Why/why not?

    b) What extra or alternative resources would you need to make it feasible?

6) Is there anything more you would like to add?
